# Disrupted Intestinal Microbiota and Intestinal Inflammation in Children with Cystic Fibrosis and Its Restoration with Lactobacillus GG: A Randomised Clinical Trial

**DOI:** 10.1371/journal.pone.0087796

**Published:** 2014-02-19

**Authors:** Eugenia Bruzzese, Maria Luisa Callegari, Valeria Raia, Sara Viscovo, Riccardo Scotto, Susanna Ferrari, Lorenzo Morelli, Vittoria Buccigrossi, Andrea Lo Vecchio, Eliana Ruberto, Alfredo Guarino

**Affiliations:** 1 Department of Translational Medical Science, Section of Pediatrics, University Federico II, Naples, Italy; 2 Centro Ricerche Biotecnologiche, Università Cattolica del Sacro Cuore, Cremona, Italy; Charité-University Medicine Berlin, Germany

## Abstract

**Background & Aims:**

Intestinal inflammation is a hallmark of cystic fibrosis (CF). Administration of probiotics can reduce intestinal inflammation and the incidence of pulmonary exacerbations. We investigated the composition of intestinal microbiota in children with CF and analyzed its relationship with intestinal inflammation. We also investigated the microflora structure before and after *Lactobacillus* GG (LGG) administration in children with CF with and without antibiotic treatment.

**Methods:**

The intestinal microbiota were analyzed by denaturing gradient gel electrophoresis (DGGE), real-time polymerase chain reaction (RT-PCR), and fluorescence *in situ* hybridization (FISH). Intestinal inflammation was assessed by measuring fecal calprotectin (CLP) and rectal nitric oxide (rNO) production in children with CF as compared with healthy controls. We then carried out a small double-blind randomized clinical trial with LGG.

**Results:**

Twenty-two children with CF children were enrolled in the study (median age, 7 years; range, 2–9 years). Fecal CLP and rNO levels were higher in children with CF than in healthy controls (184±146 µg/g vs. 52±46 µg/g; 18±15 vs. 2.6±1.2 µmol/L NO_2_^−^, respectively; *P*<0.01). Compared with healthy controls, children with CF had significantly different intestinal microbial core structures. The levels of *Eubacterium rectale*, *Bacteroides uniformis*, *Bacteroides vulgatus*, *Bifidobacterium adolescentis*, *Bifidobacterium catenulatum*, and *Faecalibacterium prausnitzii* were reduced in children with CF. A similar but more extreme pattern was observed in children with CF who were taking antibiotics. LGG administration reduced fecal CLP and partially restored intestinal microbiota. There was a significant correlation between reduced microbial richness and intestinal inflammation.

**Conclusions:**

CF causes qualitative and quantitative changes in intestinal microbiota, which may represent a novel therapeutic target in the treatment of CF. Administration of probiotics restored gut microbiota, supporting the efficacy of probiotics in reducing intestinal inflammation and pulmonary exacerbations.

**Trial Registration:**

ClinicalTrials.gov NCT 01961661

## Introduction

Culture-independent, molecular and metagenomic techniques have provided new insights into the complex interactions between mammalian hosts and their gut microbial species [1]. It is increasingly evident that gut microbes influence the host’s metabolic and immunological activities [2], [3]. Accordingly, intestinal microorganisms may influence the development of both intestinal and generalized inflammation, although the mechanism remains unclear [4], [5].

In patients with cystic fibrosis (CF), the intestine is a site of inflammatory processes. Several biomarkers, including calprotectin (CLP), S100A12, IL-8, IgM, IgG, neutrophil elastase, tumor necrosis factor-α, and eosinophil cationic protein, have been used to detect intestinal inflammation in patients with CF [6]. We previously found increased concentrations of fecal CLP and rectal nitric oxide (rNO) in children with CF, suggesting that intestinal inflammation is a feature of CF [7]. This finding was confirmed by endoscopy in children with CF [8].

Recently, it was reported that patients with CF have a different pattern of intestinal microbes than do healthy controls [9]–[10]. The majority of bacteria present in the intestinal and respiratory tracts were represented by 8 distinct genera, and there was a microbial link between the two organ systems that was reflected in core microbiota dominated by *Veillonella* and *Streptococcus* species [11]. Further, fecal samples from CF patients consistently demonstrated reduced loads of bifidobacteria [12].

In a preliminary cross-over trial in children with CF, administration of *Lactobacillus rhamnosus* GG (LGG) was associated with a reduction in intestinal inflammation as measured by CLP and rNO concentrations [7]. Interestingly, administration of LGG was associated with a reduction in pulmonary exacerbations and hospital admissions and an improvement in the forced respiratory volume in 1 s (FEV_1_) [13], [14]. These data suggest that probiotics delay respiratory impairment, which is a major goal in the treatment of CF.

On the basis of these findings, we hypothesized that there exists a link between the composition of intestinal microbiota and inflammation. Although direct evidence is not available, the intestinal microenvironment in children with CF is expected to be different from that of healthy children as a consequence of Cystic fibrosis transmembrane conductance regulator (CFTR) malfunction, frequent use of antibiotics, pancreatic enzyme supplementation, and pharmacological suppression of gastric acid production. Intraluminal pH varies with the anatomical site and microbial fermentation of dietary residues; these variations may affect the composition of the bacterial community and its metabolic activity [15]. Interestingly, among children with atopy, the administration of LGG induced a global modification of intestinal microflora, suggesting that an intestinal microbiome composed of a multitude of probiotic species may protect against the development of allergic disease when compared with one dominated by a single commensal species [16].

The aims of the present study were 1) to investigate the composition of gut microbiota in children with CF as compared with healthy controls, 2) to investigate the correlation between microbial balance and intestinal inflammation, and 3) to test the hypothesis that LGG administration restores intestinal microflora and decreases inflammation in children with CF. First, we analyzed fecal microbiota by carrying out denaturing gradient gel electrophoresis (DGGE) and real-time polymerase chain reaction (RT-PCR) of fecal samples of children with CF. To investigate the effects of the administration of LGG on composition of microbiota and intestinal inflammation, 22 children with CF were enrolled in a short-term study with administration of LGG. The effect of probiotic intake on intestinal microbial structure was investigated by fluorescence *in situ* hybridization (FISH) based on the results of DGGE and RT-PCR.

## Patients and Methods

### Study Design

The protocol for this trial and supporting CONSORT checklist are available as supporting information; see Checklist S1 and Protocol S1.

We enrolled clinically stable children with CF who had no acute intestinal or extraintestinal diseases and had not taken antibiotics for at least two weeks. The following parameters were recorded–sex, age, body weight, CF genotype, and severity of mutations. CF genotype was classified as severe if 2 severe mutations were present, and mild if at least 1 mutation was mild, according to the mechanisms by which the mutations disrupt CFTR function [17].

Intestinal inflammation and the composition of intestinal microbiota were investigated. Stool samples were collected from healthy controls in order to investigate intestinal microbiota composition in an age-matched population. Intestinal microflora was also evaluated by FISH in an additional population of children with CF who were on antibiotic treatment. Twenty-two children with CF were enrolled in the randomized double-blind clinical trial in order to evaluate the effect of LGG on intestinal inflammation and intestinal microflora composition.

Patients were randomly assigned to the treatment or placebo arms. Patients randomized to the treatment arm received 6×10^9^ CFU of LGG once daily for 1 month. Patients randomized to the placebo arm received placebo once daily for 1 month. The randomization protocol was generated by a statistician not involved in patient management. LGG and placebo formulations were masked and coded appropriately to ensure blindness. LGG formulations were composed of maltodextrin (163 mg), gelatin capsule (75 mg), LGG (60 mg), and magnesium stearate (2 mg). The placebo was composed of maltodextrin (223 mg), gelatin capsule (75 mg), and magnesium stearate (2 mg). LGG and placebo capsules were identical in appearance, taste, and color, and only a numeric code differentiated the two formulations.

Before and after administration of LGG or placebo, a microbiological investigation was carried out using FISH, and intestinal CLP concentration was measured. This study constituted a pilot study in preparation for a large trial, and it was formally registered as a clinical trial at clinicaltrials.gov (NCT 01961661). The Ethical Committee of University Federico II of Naples approved the protocol. Written informed consent was obtained for each enrolled child from the parents.

### Intestinal Inflammation

Intestinal inflammation was investigated by fecal CLP and rNO production. Fecal CLP was measured by an enzyme-linked immunosorbent assay (Calprest Eurospital SpA, Trieste, Italy). Normal values for fecal CLP were 0–50 µg/g of stool; values of 50–100 µg/g were considered intermediate and required follow-up, and values >100 µg/g indicated intestinal inflammation [18].

The stable NO metabolites NO_2_^−^ and NO_3_^−^ were measured by the Griess reaction. rNO levels measured in healthy age-matched children were used as the normal reference [19].

### Bacterial DNA Isolation

A total of 50 mg of fecal material was incubated at 37°C in lysozyme buffer (100 mM Tris-HCl, pH 8, 25% sucrose, 10 mM EDTA, and 10 mg/mL of lysozyme) for 1 h and then processed by the Maxwell® 16 System (Promega, Madison, WI, USA) using the Maxwell® 16 DNA purification kit. The extracted bacterial DNA was eluted in 400 µL of elution buffer and stored at −80°C.

### DNA Amplification

The total bacterial DNA extracted from feces was used as a template in the PCR reactions. The V_2_–V_3_ region of the 16S rRNA gene was amplified using universal primers Hda1-GC and Hda2 [20].

Amplicons for DGGE analysis of the *Bacteroides*-*Prevotella* group were obtained using the primers Bac303F and Bac708R [20]. The 418-bp amplification product obtained was used as a template in nested PCR with primers Hda1-GC and Hda2 [21].

### DGGE Analysis

All DGGE analyses were performed using the INGENYphorU-2x2 system (INGENY International, Amundsenweg, The Netherlands). Amplicons were analyzed on 8% polyacrylamide (40% acrylamide-bis, 37.5∶1) gel with a 40% to 65% denaturing gradient of urea and formamide increasing in the direction of electrophoresis. The gel was run with a constant voltage of 80 V at 60°C for 18 h in 1× Tris-acetate buffer (pH 8.0).

The Fingerprinting II software([Bio-Rad, Hercules CA, USA) was used to compare DGGE profiles using the Pearson’s similarity coefficient for comparison and the UPGMA algorithm for the dendrogram construction. The Quantity One software([version 4.6.0; Bio-Rad, Hercules CA, USA) was used for the identification and quantification of bands.

### Recovery of Amplicons and Sequencing

Bands were cut from DGGE gels, eluted at 4°C for 24 h, and re-amplified according to the PCR conditions described above. The PCR products were purified using Wizard Plus® SV Minipreps-DNA Purification System (Promega) and were sequenced (BMR Genomics, Padova, Italy). Sequences obtained were analyzed using NCBI BLAST (Basic Local Alignment Search Tool) and Ribosomal Database Project (RDP).

### RT-PCR

Copy numbers of the 16S rRNA gene from *Bacteroides*/*Prevotella* group, *Eubacterium rectale*, *Bacteroides vulgatus*, and *Bifibobacterium* were determined using previously reported primers [21]–[24]. Quantification was carried out in triplicate using the Light Cycler System (Roche Applied Science, Monza, Italy). Primer concentrations in the reaction mixture were 300 nM for *B. vulgatus*, *E. rectale*, and *Bifibobacterium* and 500 nM for the *Bacteroides*/*Prevotella* group.

*Escherichia coli* and *Bacteroides uniformis* were quantified using the LightCycler® TaqMan® Master mix (Roche Applied Science, Mannheim Germany). The primers and probe used to quantify *E. coli* were described by Scupham [22], and those for *B. uniformis* were published by Penders *et al*. [24].

Standard curves were obtained using reference DNA provided by the American Type Culture Collection (ATCC). Purified and quantified genomic DNA used were *Bifidobacterium infantis* ATCC 15697D, *B. vulgatus* 8482D-5, and *E. coli* ATCC 700926D-5. Genomic DNA of *E. rectale* DSM 17629, *B. uniformis* DSMZ 6597, *Bifidobacterium catenulatum* LMG 11043, *Bifidobacterium adolescentis* DSMZ 20083, and *Faecalibacterium prausnitzii* DSMZ 17677 was extracted from 5 mL of activated culture using the Genomic DNA extraction Kit [Promega] and quantified with a Qubit™ fluorometer (Invitrogen, Milan, Italy).

A standard curve was obtained by 10-fold dilutions of genomic DNA for each reference genomic DNA. The number of genomes was calculated considering the genome size; the differences in RNA copy numbers were excluded.

### Sample Collection and Handling for FISH Evaluation

FISH was performed on fecal samples collected from 22 clinically stable children with CF, 20 age-matched healthy controls, and 10 children with CF on antibiotic treatment. Clinically stable children with CF were then treated with LGG([6×10^9^ cfu/day) or placebo for 4 weeks, and intestinal microbiota was evaluated immediately after administration. Based on the results obtained from DGGE and RT-PCR, we used FISH to investigate the presence of *Bacteroides* and *E. rectale* since both species were consistently reduced in children with CF. *F*. *prausnitzii* was chosen, because it has been associated with the pathogenesis of inflammatory bowel diseases [25].

Fecal samples were fixed in Carnoy’s solution and embedded in paraffin, cut longitudinally into 4-µm sections, placed on SuperFrost slides (Thermo Scientific, Italy) [26], and incubated with hybridization solution (20 mM Tris-HCl, 0.9 M NaCl, 0.1% SDS, and 1% formamide, pH 7.4) with EUB mix positive control oligonucleotide probes (100 ng of EUB I, EUBII, and EUBIII). For *Bacteroides*, *E. rectale*, and *F. prausnitzii*, the slides were incubated with hybridization buffer with 25 ng of their respective FISH probes for 45 min at 50°C and visualized using a Nikon 80i Eclipse epifluorescence microscope with a Nikon DS-U2 color camera and NIS-Elements imaging software (Nikon, Tokyo, Japan). Bacteria were quantified by count. The oligonucleotide probes used in this study were synthesized by MWG Eurofins (MWG Operon Ebersberg, Germany). All samples were analyzed with the Eub338 mix probe conjugated with fluorescein isothiocyanate (FITC, green signal) at the 5′ end (positive control that reacts with all bacteria) [27], and a species-specific probe was conjugated with a single fluorescent carbocyanine molecule (Cy3, red signal).

### Statistical Analysis

Statistical analysis was performed using GraphPad Instat 3 software. A *t*-test was used to evaluate the differences in mean fecal CLP, fecal rNO, and counts of bacterial species between CF patients and age-matched healthy controls. To evaluate whether there is a possible link between intestinal inflammation and abnormalities in microbiota, patients were grouped by normal or abnormal fecal CLP concentration. The difference in the mean number of bands in the two groups was evaluated by *t*-test. The Pearson’s correlation coefficient was used to investigate the correlation between intestinal inflammatory markers and the richness of intestinal microbiota, as determined by the number of bands during DGGE analysis and the specific bacterial counts (amount of bacteria by RT-PCR).

The biological diversity of microbiota in children with CF and healthy controls was estimated by the number of bands and their intensity via DGGE. The Shannon-index (H’) was used to evaluate the richness of bacterial species, as it reflects the abundance of microbial species [28]. The similarity of DGGE profiles within and between groups was analyzed by the similarity coefficient. The number of bands and the Shannon index of the two groups were uniformly distributed, and the Student’s *t*-test was used to evaluate the difference.

RT-PCR results are expressed as means ± SD; logarithms of the number of genomes were used if required to achieve normal distributions. Data were subjected to analysis of variance by using the GLM procedure of SAS (SAS for Windows, version 9.1). Statistical significance was set at *P* ≤ 0.05.

## Results

### Study Population

Twenty-two children with CF were enrolled (13 males, median age of 7 years, range 2–9 years). Twelve children were homozygous, and 10 children were heterozygous for the ΔF508 mutation. Only 1 patient had a mild genotype. All enrolled patients were in stable clinical condition at the time of enrolment, as indicated by the absence of exacerbations, lack of hospitalization, and lack of treatment with steroids or antibiotics for at least two weeks before enrolment. Subjects were not receiving probiotics.

### Intestinal Inflammation

The mean fecal CLP concentration was significantly higher in children with CF than in age-matched healthy controls (184±146 µg/g vs. 52±46 µg/g; *P*<0.01). Fecal CLP concentration was measured in 19 children with CF. Increased fecal CLP concentration (>100 µg/g) was measured in 63% of children with CF (12/19 subjects).

**Figure 1:**
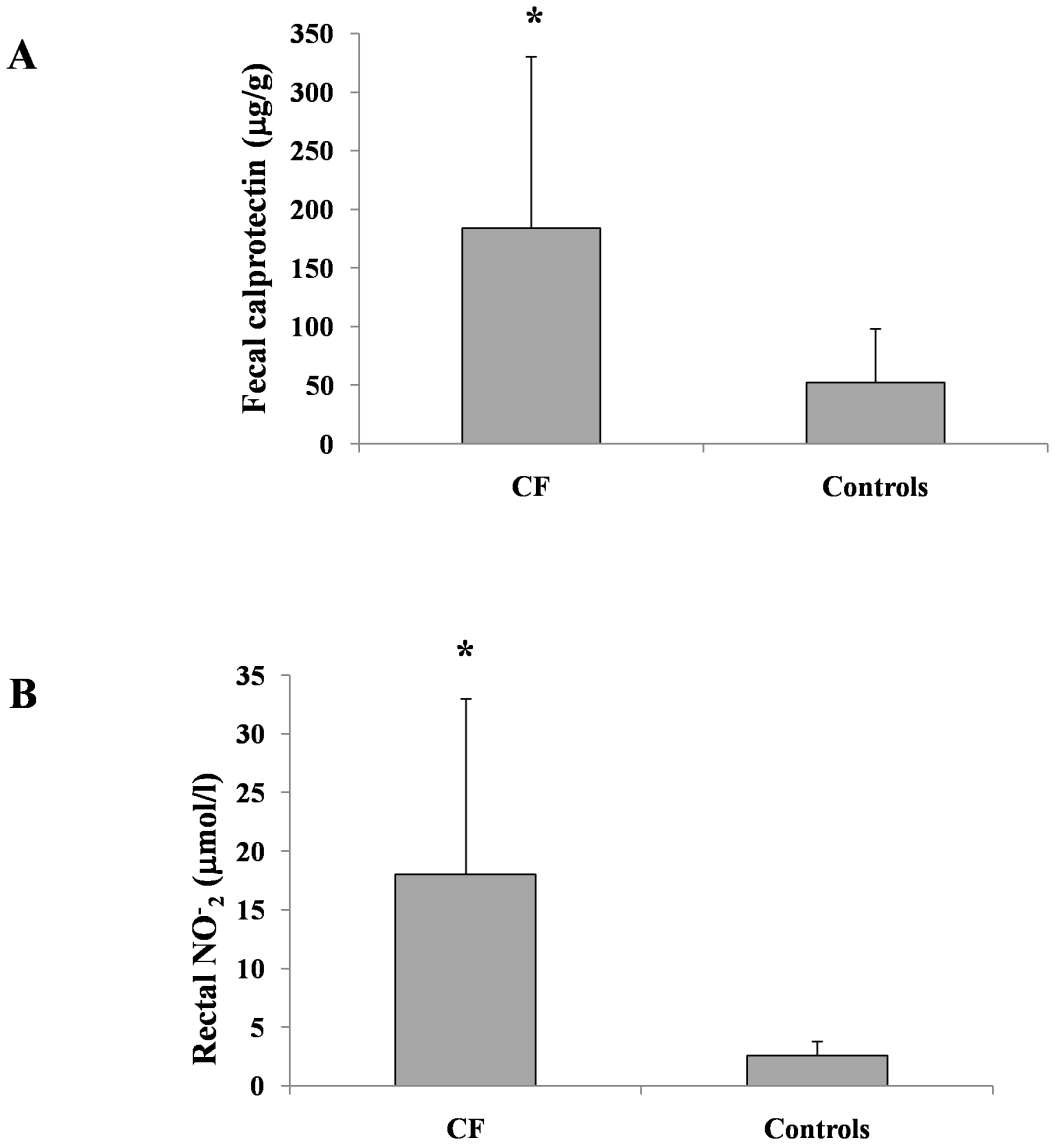
Intestinal inflammation markers in CF and healthy children. Fecal calprotectin (A) and rectal nitric oxide (B) concentrations in children with CF and in healthy controls (*P*<0.01).

rNO concentration was measured in 12 of the 22 children with CF. The mean rNO concentration was significantly higher in children with CF than in healthy controls (18±15 vs. 2.6±1.2 µmol/L NO_2_; *P*<0.01; Figure 1). The majority of children with CF (11/12) had increased concentrations of rNO, similar to our previous results [7].

### DGGE Analysis

**Figure 2:**
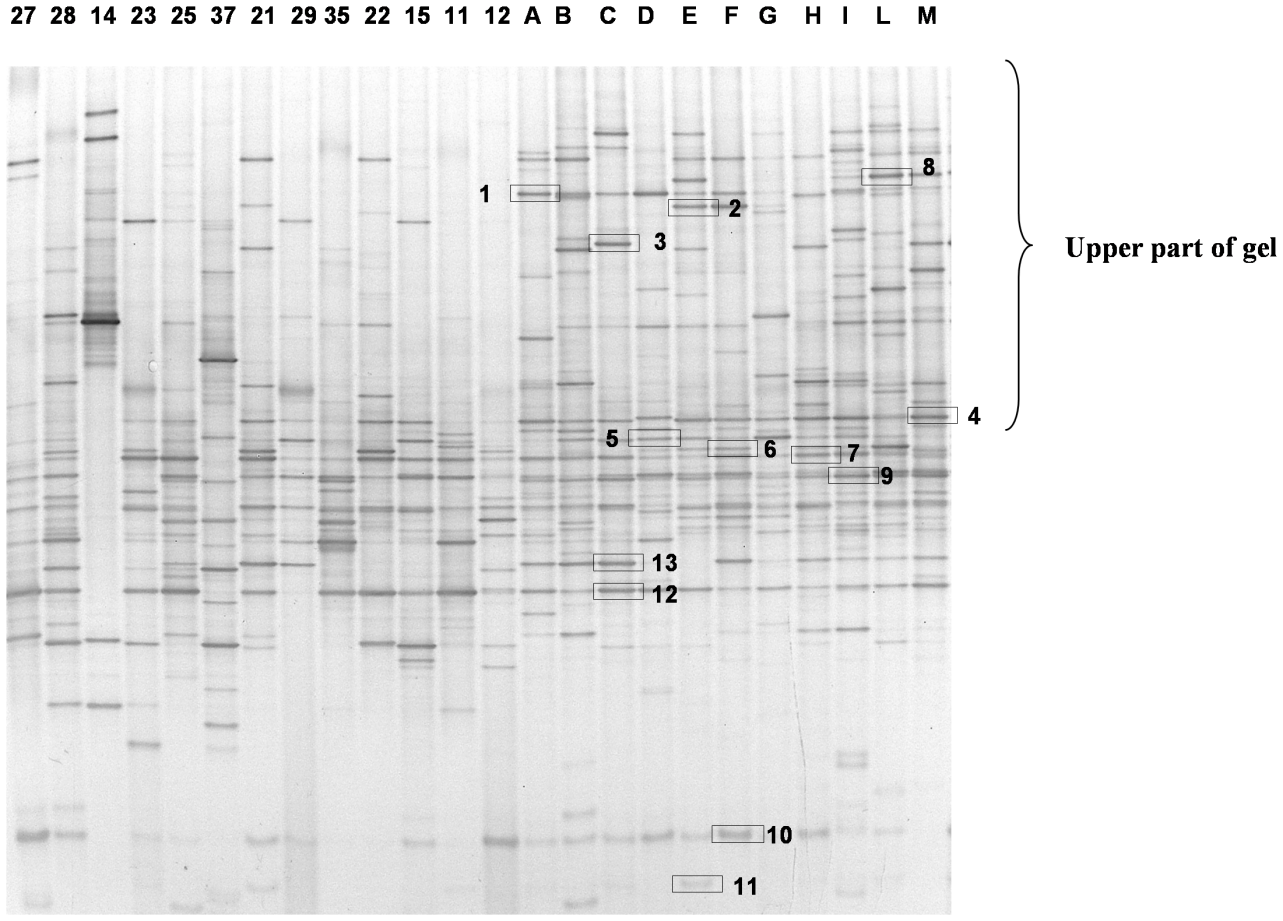
Microbial diversity in CF and healthy children. DGGE profiles of intestinal microbiota in children with CF (indicated by numbers) and age-matched healthy controls (indicated by letters) obtained with universal Hda1-Hda2 primers.

**Figure 3:**
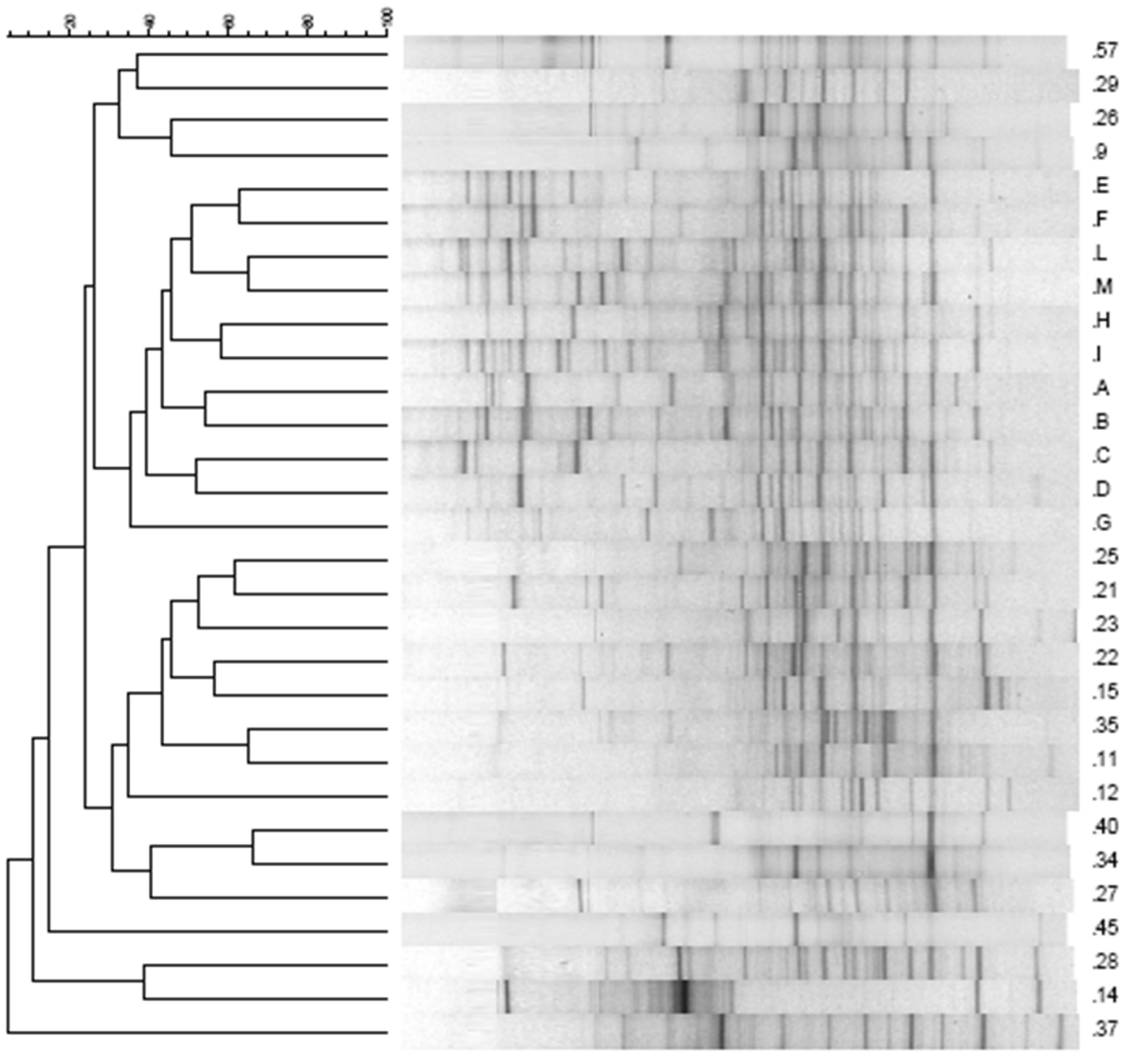
A hierarchical cluster analysis of the banding patterns in CF and healthy children. Dendrogram constructed using Pearson’s correlation coefficients and the UPGMA algorithm of DGGE gels obtained analyzing the V2–V3 region of all fecal samples. Gels were analyzed using Fingerprinting II software (Bio-Rad). Healthy subjects are indicated with capital letters, and CF patients with numbers.

The dominant fecal bacteria among children with CF and healthy subjects were determined by DGGE analysis using Hda1-GC-Hda2 primers targeting the V2–V3 region of the 16S rRNA. The DGGE profile is shown in Figure 2. The DGGE profiles significantly differed between the two groups. Compared to controls, children with CF showed a reduced number of bands, especially in the upper part of the gel. DGGE profiles from healthy subjects appeared richer and more homogeneous than those from children with CF. The DGGE profiles from healthy controls showed a common core with similar bands detected in all samples despite slight differences in intensity. There was no such common core among children with CF whose profiles were variable, as judged by the number of samples in which common species were detected (Table 1). The analysis of DGGE gels by Fingerprint II software supported the existence of two different clusters of profiles. A dendrogram was generated from the comparison of samples of children with CF and healthy controls (Figure 3).

**Table 1:** Sequence analysis of the bands indicated in Figure 2.

Bandnumber	% of blastsimilarity	Nearest species	Accessionnumber	Presence in profilesof healthy controls (%)	Presence in profilesof children withCF (%)
1	100	*Roseburia faecis*	NR 042832	80	7.69
2	99	*Ruminococcus bromii*	L76600	27	0
3	100	*Ruminococcus gnavus*	NR036800	18	0
4	100	*Eubacterium rectale, Pseudobutyrrivibrio ruminis*	AY804151 NR026315	100	53.85
5	100	*Roseburia faecis*	AY305310	23	42.86
6	97	*Ruminococcus bromii*	NR025930	100	36.36
7	100	*Robinsoniella peoriensis Hespellia porcina Lactonifactor longoviformis*	AF445285 AF445239 NR043551	100	53.84
8	99	*Ruminococcus gauvreauii Coprococcus comes Ruminococcus torques*	NR044265 EF031542 L76604	18.18	0
9	100	*Faecalibacterium prausnitzii*	AJ413954	100	15.38
10	100	*Bifidobacterium catenulatum B. pseudocatenulatum*	NR041875 NR037117	90	53.85
11	100	*Bifidobacterium adolescentis*	GQ227713	36.36	15.38
12	100	*Faecalibacterium prausnitzii*	AJ413954	100	69.23
13	100	*Robinsoniella peoriensis Hespellia porcina Lactonifactor longoviformis*	AF445285 AF445239 NR043551	72.72	7.69

The differences in the number of bands and in the Shannon index (H’) between children with CF and healthy controls were used to qualitatively analyze the diversity of fecal microbial communities. The analysis revealed significant differences between the two groups (Table 2). The mean number of bands in the healthy controls was 26.73±2.87 as compared with 16.00±4.54 in children with CF (*P*<0.001). The Shannon index was 2.13±0.30 in healthy controls and 1.19±0.66 in children with CF (*P*<0.0001).

**Table 2:** Analysis of the V2–V3 region of DGGE profiles among children with CF and healthy controls.

Group	Diversity of microbiota by DGGE analysis		Analysis of the upper part ofthe DGGE gels		Microbiota similarity	
	DGGE bands (mean ± SD)	Shannon index (mean *H’* ±SD)	DGGE bands (mean ± SD)	Shannon index (mean *H’*±SD)	Similarity coefficientswithin profiles of subjectsof the same group	Similarity coefficients within profiles of subjects of the two groups
Healthy controls	26,73±2,87	2,13±0,30	9,55±2,34	3,21±0,16	43.08±9.30	28.41±17.7
CF	16,00±4,54	1,19±0,66	3,95±2,93	2,55±0,27	24.28±17.45	
*P*-value	<0.0001	<0.0001	<0.0001	<0.0001	<0.0001	0.000

**Figure 4:**
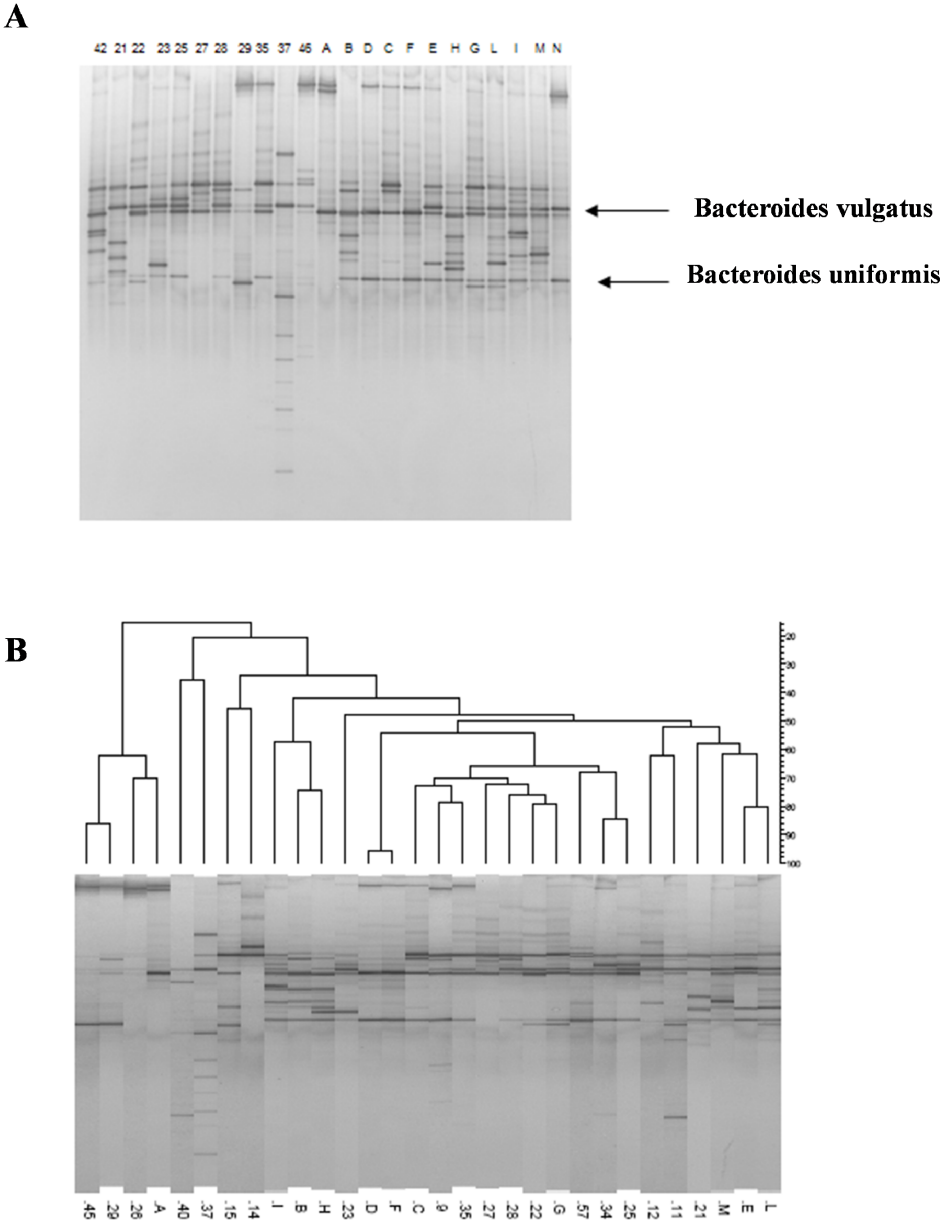
DGGE profiles of *Bacteroide*/*Prevotella* group in CF and healthy children. A, Nested DGGE of *Bacteroide*/*Prevotella* group profiles in children with CF (indicated by numbers) and age-matched healthy controls (indicated by letters). The bands corresponding to *Bacteroides vulgatus and Bacteroides uniformis* are reduced in children with CF as compared with healthy controls. B, Dendrogram of DGGE gel obtained using primers for analysis of *Bacteroides*/*Prevotella* group.

Numbers of bands (means) and Shannon index (H’) obtained from DGGE profiles of the two groups. The number of bands and Shannon index were calculated for each DGGE profile and for a portion of it. The upper part of the gel corresponds to the upper part of the profile arbitrarily chosen for analysis, as shown in Fig 4. Evaluation of the similarity between microbiota of children with CF and health controls is presented as the mean of Pearson’s similarity coefficients within and between groups. *P* values of <0.05 were considered statistically significant.

The upper part of the gel was analyzed separately from the lower part, because this area contained very few bands in children with CF as compared with healthy controls (Figure 3).

A comparative analysis of the similarities among all DGGE profiles from fecal samples of children with CF and healthy controls revealed a broad range of similarity coefficients for DGGE profiles (0.00–66.31 for children with CF and 21.39–65.35 for healthy controls). The mean similarity values of total bacteria, calculated using the Hda1GC-Hda2 DGGE profiles, indicated reduced biological variability in children with CF (24.28±17.45 bands) as compared with healthy controls (43.08±9.30 bands, *P<*0.0001, Table 2).

The DGGE profile of healthy controls was characterized by two major bands with high sequence similarity to *B. vulgatus* and *B. uniformis*. These two amplicons were detected in few children with CF (Figure 4A). The dendrogram obtained by analyzing the DGGE gels of the *Bacteroides/Prevotella* group is shown in Figure 4B.

The bands isolated from the Hda1GC-Hda2 DGGE profile of healthy controls exhibited a high degree of similarity with E. rectale/Pseudobutyrivibrio ruminis, Roseburia faecis, Ruminococcus bromii, Robinsoniella peoriensis/Hespellia porcina/Lactonifactor longoviformis, F. prausnitzii, and Bifidobacterium pseudocatenulatum/B. catenulatum. Many of the species detected in the healthy controls were not detected in the majority of children with CF; in particular, R. bromii was never detected in children with CF. Table 1 shows the identification of the bands isolated from DGGE gels and their frequency in children with CF and healthy controls.

### RT-PCR Analysis

**Figure 5:**
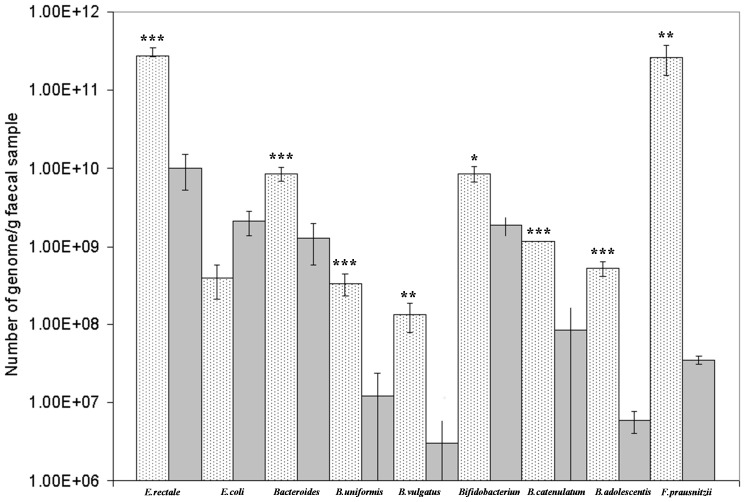
RT-PCR analysis of selected bacterial species in stools of CF and healthy children. Quantitative differences in *E*. *rectale, E*. *coli, Bacteroides*/*Prevotella group, B*. *uniformis, B*. *vulgatus, Bifidobacterium* spp., *B*. *catenulatum* group, *B. adolescentis*, and *F*. *prausnitzii* as determined by RT-PCR in children with CF and healthy individuals. Values are means ± SD. Differences indicated with *** are extremely statistically significant (*P*<0.0001); differences indicated with ** are very statistically significant (*P = *0.003), and differences indicated with * are statistically significant (*P = *0.03).

To confirm the qualitative data obtained using DGGE, a quantitative analysis was performed using RT-PCR. As shown in Figure 5, children with CF had a significant reduction in *E. rectale* and *Bacteroides* spp., mainly *B. vulgatus* and *B. uniformis*, as compared with healthy controls. Moreover, the loads of *B. catenulatum* group, *B*. *adolescentis*, and *F. prausnitzii* were significantly higher in the healthy controls as compared with children with CF. *E. coli* was more abundant in children with CF as compared with healthy controls, but the difference was not statistically significant.

### Relationship between Intestinal Inflammation and Abnormalities in Intestinal Microbiota

We used a Pearson’s correlation to evaluate the relationship between composition of intestinal microbiota and intestinal inflammation. There was no statistically significant correlation between the total number of bands and fecal CLP concentration. When subjects were grouped according to fecal CLP, the mean number of bands was higher, although not significantly, in children with fecal CLP<100 µg/g as compared with children with fecal CLP>100 µg/g (18±5 vs. 14±4; *P* = 0.063). This finding suggests a link between intestinal inflammation and reduced biodiversity of intestinal microbiota. There was a significant correlation between the number of bands in the upper part of the gel and fecal CLP concentration (r = 0.53; *P = *0.018). The was no correlation between the number of individual bacterial species and fecal CLP concentration in children with CF. Furthermore, there was no correlation between rNO concentration and any specific microbial pattern.

### Evaluation of Intestinal Microbiota before and after LGG Administration

We next performed a randomized, double-blind clinical trial to further investigate the composition of intestinal microflora in children with CF and to directly test the hypothesis that probiotic administration restores intestinal microflora. Therefore, we administered LGG or placebo for 4 weeks to 22 children with CF (LGG, n = 10; placebo, n = 12). We investigated the composition of intestinal microbiota before and after LGG or placebo administration by FISH. Further, we compared the results with those from age-matched healthy controls and children with CF treated with antibiotics.

**Figure 6:**
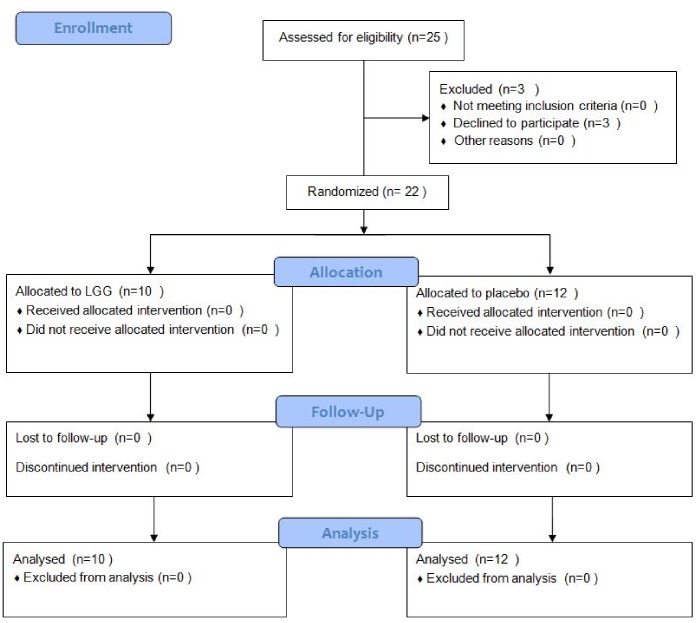
Flow diagram of enrolled patients.

**Figure 7:**
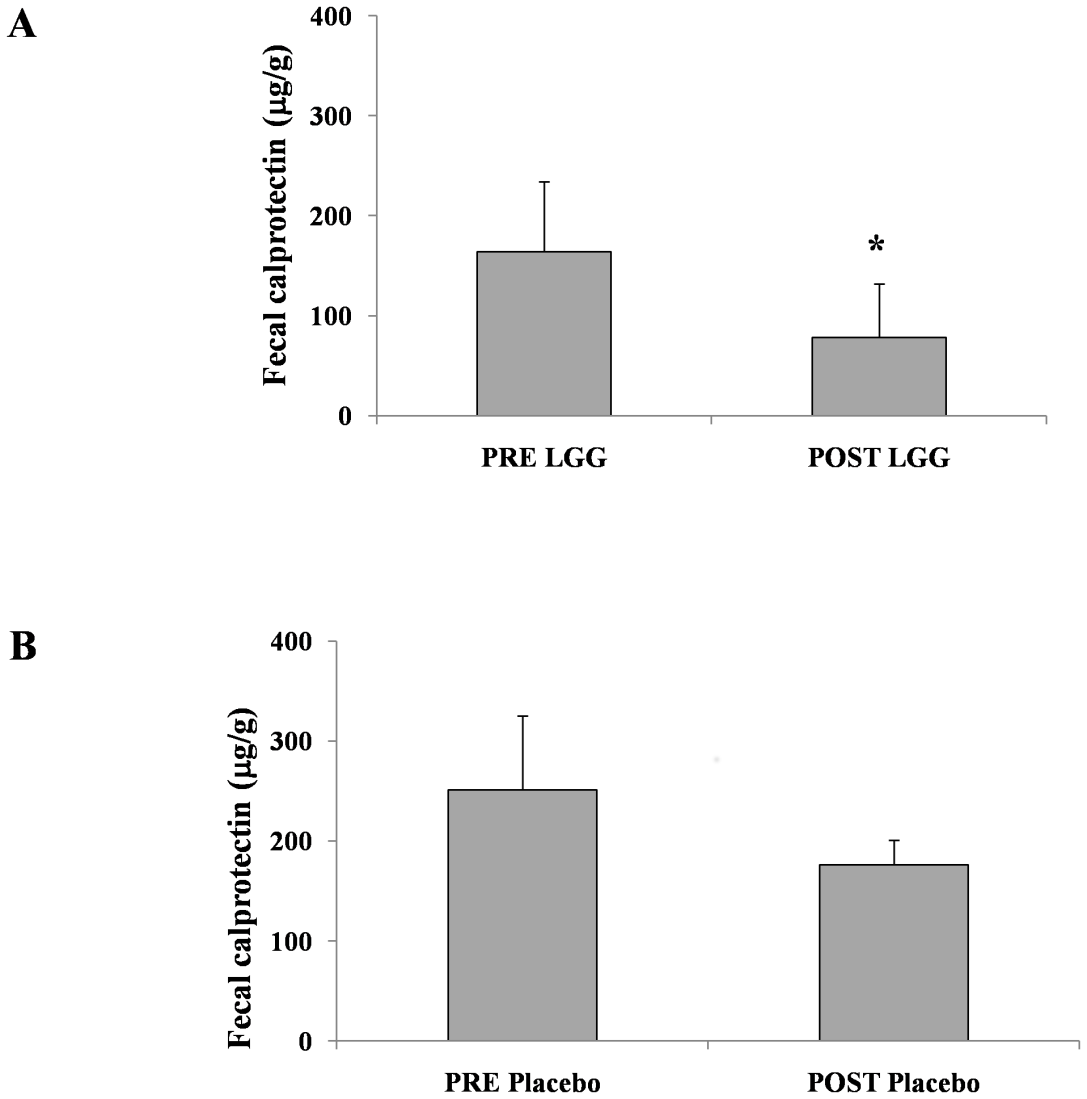
Quantitative analysis of different bacterial species by FISH in children with CF, healthy controls, and children with CF after antibiotic treatment. A significant reduction in the levels of *Bacteroides* spp., *Eubacterium rectale*, and *Fecalibacterium prausnitzii* spp. was observed in children with CF as compared with that in healthy controls. A further reduction in the number of *Bacteroides* spp. and *E. rectal*e was observed in children with CF after antibiotic treatment as compared with that in children with CF who were not treated with antibiotics. *P<0.001; **P<0.01.

The flow diagram of enrolled children is presented in Figure 6. Using DGGE, we identified *Bacteroides, F. prausnitzii*, and *E. rectale* as biomarkers of microbial disruption and selected specific primers for FISH analysis. The bacterial loads of *Bacteroides, F. prausnitzii*, and *E. rectale* were significantly reduced in children with CF as compared with healthy controls (2.5±0.6 vs. 13.8±2.3×10^10^/mL, *P*<0.001; 2.3±0.7 vs. 13.4±3.1×10^10^/mL, *P<*0.001; 4.9±1.1 vs. 11.1±1.3 10^10^/mL, *P*<0.001, respectively) (Figure 7). These data confirm the results obtained by DGGE and RT-PCR. Further, a significant reduction in *Bacteroides and E. rectale* was found in children with CF who were treated with antibiotics as compared with children with CF children who were not treated with antibiotics (2.5±0.6 vs. 0.3±0.3×10^10^/mL, *P*<0.01; 4.9±1.1 vs. 0.8±0.5×10^10^/mL, *P*<0.01, respectively). In children with CF there was also a reduction in *F. prausnitzii* counts that did not reach statistical significance (Figure 7).

Next, we investigated whether administration of LGG could restore composition of microbiota in children with CF. Children with CF treated with LGG had a significant increase in *Bacteroides* counts as compared with those treated with placebo (Table 3). There was a non-significant increase in the counts of *F. prausnitzii* in children with CF treated with LGG as compared with children with CF treated with placebo. There was no change in *E. rectale* counts after treatment with LGG (Table 3).

**Table 3:** Bacterial counts before and after LGG or placebo administration in children with CF as assessed by FISH.

Bacterial species	*Bacteroides*	*F. prausnitzii*	*E. rectale*
Before LGG (n = 10)	1.7 8±0.47×10^10^/mL	2.31±0.98×10^10^/mL	4.23±1.17×10^10^/mL
After LGG	6.62±1.33×10^10^/mL*	4.77±1.3×10^10^/mL	4.82±0.85×10^10^/mL
Before placebo (n = 12)	3.07±1.05×10^10^/mL	2.25±0.96×10^10^/mL	5.54±1.77×10^10^/mL
After placebo	4.44±1.46×10^10^/mL	2.73±1.51×10^10^/mL	11.16±2.42×10^10^/mL

*P = 0.030.

**Figure 8:**
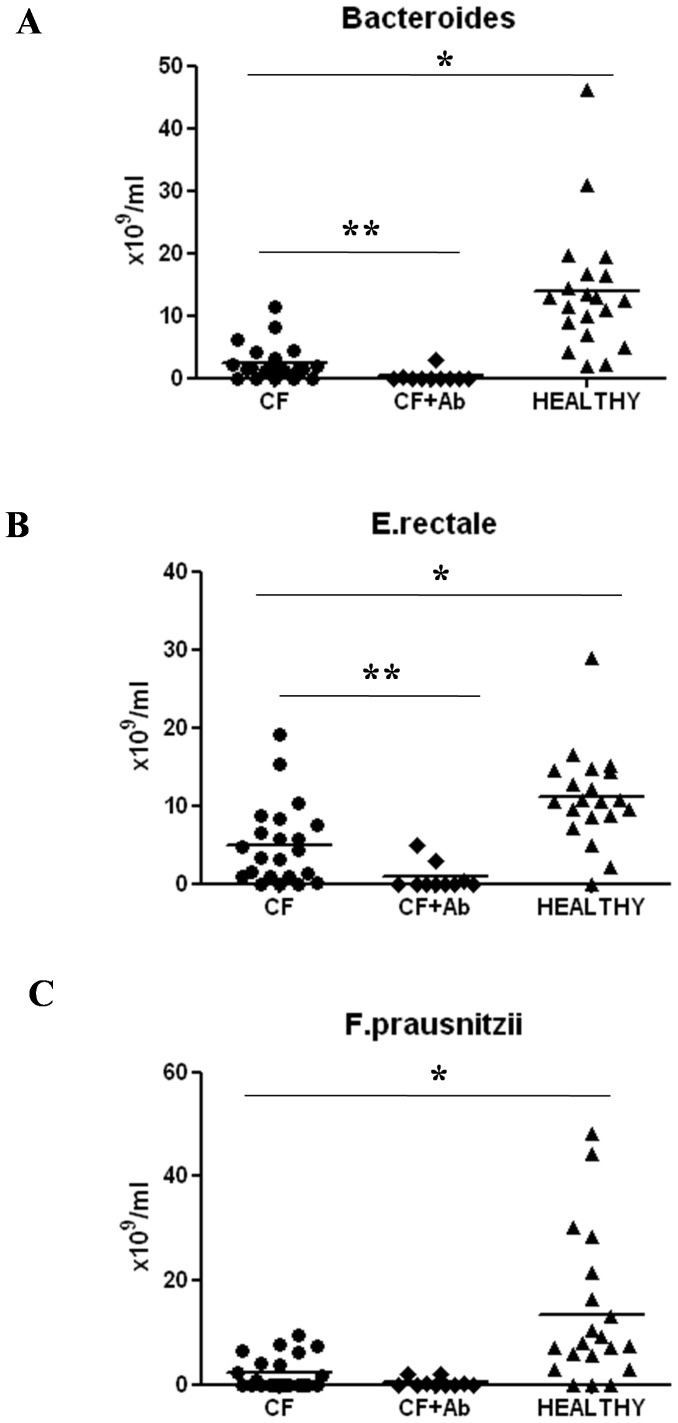
Modification of fecal CLP concentration before and after LGG or placebo treatment in children with CF. A significant reduction in fecal CLP was observed in children with CF after LGG treatment (*P<*0.05).

Intestinal inflammation was assessed by fecal CLP concentration. Children with CF who were treated with LGG showed a significant decrease in fecal CLP concentration as compared with those treated with placebo (164±70 vs. 78±54 µg/g, *P*<0.05; 251±174 vs. 176±125 µg/g, *P = *0.3, respectively; Figure 8).

## Discussion

Abnormalities in intestinal microbiota have been identified in several primary intestinal diseases, often with different patterns in adults and children. However, the causal relationship between aberrations in microbiota and specific diseases remains unclear. Specific abnormalities in intestinal microbiota that are consistently associated with a pathological condition are termed “signatures.” This raises the possibility that a specific pattern of intestinal microbiology maybe a “hallmark” of disease rather than contributes to pathophysiology of a disease [29].

We previously reported that intestinal inflammation is frequent in patients with CF [7]. The present study confirms that intestinal microecology is disrupted in children with CF and is significantly different from that of age-matched healthy controls. There were three specific hallmarks of the CF microbiota: 1) reduction in species richness, 2) reduction in species similarity among children with CF, and 3) a different pattern of bacterial species. A different pattern of bacterial species in children with CF was characterized by an absence or reduction in selected species and the presence or increase in others as compared with healthy controls.

The DGGE profiles showed a reduced number of bands, indicating a reduction in biological diversity. This result was supported by the differences in Shannon index between children with CF and healthy controls. A reduction in biodiversity of microbiota is a marker of dysbiosis and is probably a consequence of selective environmental pressures [30]. However, it is also associated with intestinal inflammation, including active inflammatory bowel disease [31]. In addition to the limited biodiversity, the common “core” of bands detected in all healthy controls was absent from children with CF. Several of the profiles of children with CF did not contain the microbiologic core, and others contained only a few bands of the core. This pattern suggests that the core structure of the intestinal microbiota is disrupted in children with CF, who may have their own specific pattern. These findings apply to children with severe CF genotypes and may not hold true in milder cases of CF.

We hypothesized that the abnormal small-intestine environment of patients with CF, which is characterized by electrolyte abnormalities and an acidic pH, could select for specific bacterial genera and species. In turn, the aberrant microbiota could contribute to, rather than result from, intestinal inflammation in CF. The hypothesis is supported by several lines of evidence. Recently, intestinal microbiota of identical twins with Crohn’s disease were molecularly analyzed; results indicated that the microbial profiles of individuals with ileal involvement significantly differed from healthy controls and from patients with colonic involvement [32]. In particular, reduced abundance of *B. uniformis* and increased abundance of *B. ovatus* and *B. vulgatus* were detected in patients with Crohn’s disease with ileal involvement as compared with those with colonic involvement and in healthy controls. *B. vulgatus* was also reduced in ileal specimens from children with Crohn’s disease, ulcerative colitis, and indeterminate colitis as compared with control subjects [33]. Low levels of *B. vulgatus*, *B. ovatus*, *B. uniformis*, and *Parabacteroides* spp. were also detected in patients with ulcerative colitis and irritable bowel syndrome as compared with healthy controls [34].

We found a similar reduction in the abundance of *B. uniformis* and *B. vulgatus* in children with CF as compared with healthy controls. We also identified a significant reduction in *E. rectale* in children with CF. *E. rectale* is a butyrate-producing bacteria, and butyrate is the preferred energy source for colonic mucosa, protecting it from colitis and colorectal cancer and promoting the normal development of colonic epithelial cells. Short-chain fatty acids (SCFAs) play a protective role against intestinal inflammation, and it is possible that SCFA-producing intestinal microbiota may prevent intestinal inflammation. We speculate that the reduced load of *E. rectale*, *B. vulgatus*, and *B. uniformis* found in children with CF may be involved in intestinal inflammation.

We also found a significant inverse correlation between the richness of CF microbiota and intestinal inflammation. This was most evident in children with fecal CLP concentrations >200 µg/g, who showed a significantly lower number of bands in the upper part of the gel, which corresponds to *Bacteroides* (data not shown). Administration of antibiotics further reduced the same microbial species already reduced in children with CF as compared with healthy controls.

We further investigated the role of probiotic administration on intestinal microbiota. Lynch *et al*. recently reported that LGG administration affected the overall composition of intestinal microbiota in a population of infants at high risk for asthma [16]. This finding raised the hypothesis that administration of a single probiotic strain may globally modify the microbiota, thus promoting bacterial communities that modulate host immune responses. In the present study, LGG administration restored the composition of intestinal microbiota, making it more similar to that of healthy controls. There was a concurrent reduction in intestinal inflammation, similar to previous trials [7], [13]. Together with previously reported benefits for respiratory exacerbations, hospital admissions, and respiratory function, our results indicate that LGG administration modifies intestinal microbiota and ultimately contributes to its beneficial clinical effects [7], [13].

In conclusion, our data provide direct evidence that intestinal microbiota are disrupted in children with CF and is associated with intestinal inflammation. Antibiotic therapy exacerbates these abnormalities. The administration of LGG may partially restore a healthy intestinal microbiota, thus promoting communities that limit intestinal inflammation and improving the disease course.

## Supporting Information

Checklist S1 **CONSORT Checklist.**
(DOC)

Protocol S1 **Trial Protocol.**
(DOC)
