# Supplementary material for: Disrupted Intestinal Microbiota and Intestinal Inflammation in Children with Cystic Fibrosis and Its Restoration with Lactobacillus GG: A Randomised Clinical Trial
Source: PLoS One. 2014 Feb 19;9(2):e87796. doi: 10.1371/journal.pone.0087796 (PMC3929570; doi:10.1371/journal.pone.0087796)
Supplement: Protocol S1 — Trial Protocol. (DOC) [file pone.0087796.s002.doc]

**Probiotics in Cystic Fibrosis**

Alfredo Guarino

Department of Pediatrics, University Federico II of Naples

**Hypothesis and specific aims.**

Cystic fibrosis (CF) is a complex, multi-system disease. Increasingly it is becoming clear that colonization of CF airways is due to the presence of a dynamic polymicrobial community that typically includes of a number of pathogenic species [1-3], and that bacterial diversity at this site is strongly influenced by host, environmental and clinical factors. We have shown that CF airway samples have more complex communities than previously appreciated. Moreover, both pediatric and adult CF airway samples possess species associated with colonization of the gastrointestinal (GI) tract e.g. *Enterococcus* spp., suggesting that this host niche may also influence the bacterial community composition of the airways. A growing body of literature and our preliminary data (see below), suggest a strong link between bacterial colonization of the GI tract, immune response and manifestations of chronic airway and GI inflammatory diseases [4, 5]. More recently several studies of both CF and non-CF patients have demonstrated a marked improvement in airway and GI inflammatory outcomes through daily supplementation with specific probiotic bacterial species [6, 7]. We have also demonstrated that the presence of *Lactobacillus casei* in high abundance (through daily supplementation) leads to a restructuring of the infant GI population, which becomes dominated by a number of known beneficial commensal bacterial members (see preliminary data). In addition, we have examined CFTR null mice and shown that their GI microbiota are structured differently and are significantly less diverse compared with wild type animals, a phenomenon we have also demonstrated in CF pediatric patients compared to healthy children (see preliminary data). Finally, previous studies have related changes in GI microbiota associated with obese individuals with increased calorific extraction from ingested food [8], demonstrating that the GI community composition governs its functionality. Given the considerable clinical involvement of the GI and respiratory tracts in CF patients, these recent studies provide a foundation for the overarching hypothesis, that altered GI microbial colonization of CF pediatrics contributes to their health status by influencing: 1. immune response leading to chronic inflammation of the GI and respiratory tracts; 2. airway colonization and 3. weight gain. Further, we hypothesize that manipulation of the GI community composition through daily feeding of a probiotic species, *L. casei*, to CF pediatrics produces a community-wide restructuring of both GI and airway microbiomes associated with improved clinical, immunological and microbiological outcomes.

To examine these hypotheses, a randomized, controlled, double blind multi-center study will be performed to determine the effect of daily supplementation of *Lactobacillus casei sps. Rhamnosus* (*Lactobacillus GG*) on GI and respiratory tract bacterial communities and to relate microbiological changes to primary and secondary clinical and immunological outcomes.

***Specific Aim 1. Define the CF gut microbiome by comparing stool samples from CF pediatric patients at baseline (prior to supplementation) to age matched healthy subjects.***

***Specific Aim 2.*** ***Determine the effect of daily Lactobacillus GG supplementation on GI bacterial communities of CF patients fed probiotic or placebo.***

**Significance.**

It is becoming clear that intestinal microflora is a complex functional unit that lives with the host in a symbiotic relationship. The gastrointestinal tract harbors more than 1000 bacterial species, whose composition is a result of several factors including age, diet, antibiotics and other drugs. The hygiene hypothesis, also known as the theory of “old friends”, implicated intestinal bacteria in the regulation of the immune response [9]. Commensal bacteria drive the immune response by inducing a maturation pattern of dendritic cells that stimulate the regulatory T-cell response. Deficient exposure to the “old friends” or changes in intestinal microecology may lead to an abnormal immune response. There is evidence that a healthy intestinal microflora protects from allergy and other immune disorders [10] by driving the development of immune response toward protection against intestinal and extraintestinal infections, including respiratory infections [11-13].

Children with CF are at risk of an abnormal intestinal microflora, as a consequence of impaired CFTR function, a heavy antibiotic load, pancreatic enzyme supplementation and acid suppression treatment [14]. Our preliminary data (unpublished, see “Preliminary studies section”) indicate that CF patients have a peculiar microbiota. Lung disease, malnutrition and pulmonary inflammation occur very early in life in people affected by CF [15].

The intestine is a site of inflammation in CF. Using immunohistochemistry and intestinal morphometric analysis, Raia et al., identified duodenal mucosal T-cell activation in the absence of alterations of villous architecture in a subset of CF children at-risk of intestinal involvement because of recurrent vomiting or chronic diarrhea who underwent upper endoscopy [16]. The T-cell response was characterized by an increased infiltration of the lamina propria by mononuclear cells expressing ICAM-1, CD25 and IFN-gamma, and by an increased number of villous enterocytes expressing transferrin receptors.

Our hypotheses are supported by preliminary data from our centre. In a subsequent study, the incidence of intestinal inflammation was investigated in a population of 30 CF children, otherwise unselected [6]. Fecal calprotectin and rectal nitric oxide concentration in rectal dialysis bags were used as non invasive markers of intestinal inflammation [6]. It was observed that intestinal inflammation was very frequent in CF. Mean calprotectin values were 4 times above normal in CF children. Nitric oxide rectal production was also increased, which supports the concept that the distal intestine represents a target organ in CF disease. In a trial with the probiotic strain *Lactobacillus rhamnosus* GG (LGG) in children with CF colonized with *Pseudomonas aeruginosa,* calprotectin and nitric oxide concentrations were decreased and values of these inflammatory marker did not return to fully normal values [6].

In a subsequent prospective, randomized, placebo-controlled, cross-over study, we investigated the incidence of pulmonary exacerbations and hospital admissions in CF children colonized with *Pseudomonas aeruginosa* [7]. Pulmonary exacerbations and of hospital admissions were fewer in patients receiving the probiotic LGG than in patients treated with oral rehydration solution (ORS). Probiotics administration also resulted in a slight increase in forced expiratory volume in 1 sec (FEV1). These data suggest that probiotics may delay respiratory impairment in CF children and support a relationship between intestinal and pulmonary inflammation.

Although the evidence available is largely preliminary and should be regarded with caution, our data implicate colonic intestinal microflora in the pathogenesis of CF, as follows:

- Colonic intestinal microflora is changed in CF as a result of changes in intestinal environmental and aggressive therapy.
- Mild to moderate intestinal inflammation is frequent in CF, at least in part, as a consequence of abnormal microflora composition.
- Administration of selected probiotics reduces intestinal inflammation and also exerts beneficial effect on intestinal microbiota .

The intestine, and specifically the intestinal microflora, may therefore be an important target for treatment in CF. The administration of probiotics is a promising treatment approach for children who are on heavy load of antibiotics.

**Experimental Design and Methods.**

***Type of study/setting***

This is a prospective, double-blind, randomized, pilot study comparing oral probiotics versus placebo. The total duration of the study will be 6 months:

- - Patients enrollment: 3 months
  - Treatment phase: 1 months
  - Data analysis and statistical evaluation: 2 months

***Study population***

**Inclusion criteria will be:**

1. A confirmed diagnosis of CF documented by sweat chloride test over 60 mmol/L and confirmed by genotype analysis with the presence of F508del/F508del or F508del/other
2. Boys and girls between 2 and 18 years of age
3. Clinical stability at enrolment, defined as no clinical evidence of acute exacerbation, no modifications in the therapeutic regimen and no hospitalization in the last 2 weeks
4. Pancreatic insufficiency
5. Basal FEV1 above 50% of predicted value

**Exclusion criteria will be:**

1. Colonization of respiratory tract with *Burkholderia cepacia* spp.
2. Steroid therapy within one month before enrolment
3. Pregnancy and fertile women taking oral contraceptives
4. Parenteral or oral antibiotics therapy within 2 weeks before enrolment
5. Regular assumption of probiotics
6. Regular assumption of azythromicin

***Study design***

Study activities are reported in the timeline (Table 1).

| **Parameters** | **Visit 1^a^** | **Visit 2^b^** |
| --- | --- | --- |
| Written informed consent | x |  |
| Randomization |  | x |
| Clinical examination | x | x |
| Genetic analysis (if not already performed) | x |  |
| Demographic | x |  |
| Anthropometric | x | x |
| Intestinal inflammation | x | x |
| Microflora |  | x |
|  |  |  |

Study design

Patients meeting the inclusion criteria will be enrolled and randomly assigned to the treatment or placebo group arm. Patients randomized to the treatment arm will receive 6x10^9^ CFU of LGG once a day for a 1-month period. Patients randomized to the placebo group will receive placebo once a day for 1 months. LGG and placebo formulations will be masked and coded appropriately to ensure blindness. LGG formulations will be composed of maltodextrin (163 mg), gelatine capsule (75 mg), LGG (60 mg), magnesium stearate (2 mg). The placebo composition will be maltodextrin (223 mg), gelatine capsule (75 mg) and magnesium stearate (2 mg). LGG and placebo capsules will be identical in appearance, taste and color and only a numeric code will differentiate the two formulations.

For these patients systemic inflammation and intestinal microflora using Fluorescent in situ Hybridization will be evaluated before starting treatment and at the end of it (0 and 1 month

***Endpoints***

Primary endpoints:

- Modification of intestinal inflammation
- Modification of intestinal microflora composition

***Methods*** .

**Intestinal inflammation**

Intestinal inflammation status will be monitored in a non-invasive manner.

At each scheduled time point, one stool sample will be collected and frozen at -20°C for fecal calprotectin analysis. Fecal calprotectin levels will be measured, using a standardized technique, in each participating center.

Fecal calprotectin concentration will be evaluated using the ELISA method (Calprest Eurospital, SpA, Trieste, Italy). Briefly, 0.1 g of feces are suspended in a buffer, pre-diluted, homogenized and centrifuged. The supernatant is collected and analyzed by an ELISA (according to the manufacturer’s instructions) with a specific polyclonal antibody. The reference values of fecal calprotectin range between 0 and 50 micrograms/g, values between 50 and 100 micrograms/g are considered intermediate, and require follow-up, whereas values over 100 micrograms/g indicate intestinal inflammation.

**Sample Collection and Handling for FISH evaluation**

FISH was performed on fecal samples collected from 22 CF children in stable clinical conditions, 20 age-matched healthy controls. Stools from 10 CF children on antibiotic treatment were also collected and analysed. CF children, in stable conditions, were then treated with LGG (6x10^9^ cfu/die) or placebo for 4 weeks and intestinal microbiota was evaluated again immediately after their administration.. Based on the results obtained by DGGE and RT-PCR, we chose to investigate the presence of *Bacteroides* and *Eubacterium rectale* since both species were consistently reduced in CF children.

*F. prausnitzii* was chosen because it has been associated with the pathogenesis of inflammatory bowel diseases.

Fecal samples, fixed in Carnoy's solution and embedded in paraffin, were cut longitudinally into 4-μm sections, placed on SuperFrost slides (Thermo Scientific, Italy) and incubated with hybridization solution (20 mM Tris–HCl, 0.9 M NaCl, 0.1% SDS and 1% formamide, pH 7.4) with EUB mix positive control oligonucleotide probes (100 ng of EUB I, EUBII and EUBIII). For *Bacteroides*, *E.rectale* and *F. prausnitzii* the slides were incubated with hybridization buffer with 25 ng of their respective FISH probes for 45 min at 50 °C and evaluated by Nikon 80i Eclipse epifluorescence microscope. Using a Nikon DS-U2 color camera and NIS-Elements imaging software (Nikon, Tokyo, Japan). Bacteria were quantified by count within a 10×10–μm area of the microscopic field representative of the region of interest. The oligonucleotide probes used in this study were synthesized by MWG Eurofins (MWG Operon Ebersberg, Germany). All samples were analyzed with the Eub338 mix probe conjugated with fluorescein isothiocyanate (FITC, green signal) at the 5′-end (positive control, which is specific for all bacteria), and an appropriate species-specific probe was conjugated with a single fluorescent carbocyanine (Cy3, red signal) molecule.

***Ethical aspects***

The study will be conducted in accordance with Good Clinical Practice (GCP). Patients will be informed about the purpose of the research, the nature of the research, responsibilities, the scientific use of the data, the right of withdrawal. They will be instructed to continue taking all currently prescribed medication and treatments, and to phone or e-mail immediately if any adverse event occurs. The investigators will evaluate the patients for any possible adverse event at each visit, using standard clinical criteria. During clinical visits patients will be segregated according to bacterial status. Clinical researchers will phone patients weekly to monitor side effects and to encourage compliance.

Written informed consent to participate to the study will be provided by the parents or legal guardian of each participant. All enrolled patients must have consented to their medical records being viewed by authorized personnel and by regulatory authorities. The protocol has been submitted and approved by the Ethics Committee of University of Naples Federico II (Coordinating Center) (protocol number 0939; approved on April 22^nd^, 2009). Potential risks for study subjects will be monitored and only for clinically relevant events, by which the patients can modify their life style changes, subjects should drop out of the study.

***Specific statistical analysis***

# *Statistical Analysis for primary endpoints*

**INTESTINAL INFLAMMATION:** The binary outcome variable is the presence of at least one intestinal inflammation episode for subject j in cluster i (i=1,..,N, j=1,…n). Based on the results of Bruzzese et al[6], a proportion of subjects with at least 1 episode of intestinal inflammation of 0.90 is expected; a reduction of 30% of such proportion is considered clinically relevant (0.90 vs 0.63). These values determine an odd of experiencing at least one intestinal inflammation episode 0.19 times smaller in the treatment group than in the control group. A intra-cluster correlation coefficient (ρ) between the two time occasions of 0.6 is assumed (this is rather a cautionary value because a smaller coefficient is expected) With a one-sided  α of 0.025 and a statistical power (1-β) of 0.80, 35 subjects per treatment group are needed to detect such difference in favour of the LGG compared to placebo after twelve months. Alternatively, by using the sample size obtained for powering the analysis of pulmonary exacerbations, all other things being equals, a power of 92% will be reached.

### *Statistical Analysis of Secondary Endpoints*

***Intestinal inflammation and fecal microflora analysis:*** a descriptive analysis will be performed. Continuous variables will be expressed as mean ± standard deviation (SD), while categorical variables as proportions. The significance of the differences between the groups will be assessed by the chi square test and Fisher’s exact test for dichotomous variables. Continuous variables will be compared with the Student t test and the Mann-Whitney test. A two-sided test was used to indicate statistical significance at a p value of <0.05.

**Bibliography.**

1. Rogers, G.B., et al., J Clin Microbiol, 2006. **44**(7): p. 2601-4.

2. Rogers, G.B., et al., Respir Res, 2005. **6**: p. 49.

3. Harris, J.K., et al., Proc Natl Acad Sci U S A, 2007. **104**(51): p. 20529-33.

4. Kalliomaki, M. and E. Isolauri, Curr Drug Targets Infect Disord, 2002. **2**(3): p. 193-9.

5. Penders, J., et al., Gut, 2007. **56**(5): p. 661-7.

6. Bruzzese, E., et al., Aliment Pharmacol Ther, 2004. **20**(7): p. 813-9.

7. Bruzzese, E., et al., Clin Nutr, 2007. **26**(3): p. 322-8.

8. Turnbaugh, P.J., et al., Nature, 2006. **444**(7122): p. 1027-31.

9. Guarner, F., et al., Nat Clin Pract Gastroenterol Hepatol, 2006. **3**(5): p. 275-84.

10. Packey, C.D. and R.B. Sartor, J Intern Med, 2008. **263**(6): p. 597-606.

11. Bruzzese, E., et al., Clin Nutr, 2009. **28**(2): p. 156-61.

12. Macpherson, A.J. and N.L. Harris, Nat Rev Immunol, 2004. **4**(6): p. 478-85.

13. Mazmanian, S.K. and D.L. Kasper, Nat Rev Immunol, 2006. **6**(11): p. 849-58.

14. Khan, T.Z., et al., Am J Respir Crit Care Med, 1995. **151**(4): p. 1075-82.

15. De Lisle, R.C., Am J Physiol Gastrointest Liver Physiol, 2007. **293**(1): p. G104-11.

16. Raia, V., et al., Pediatr Res, 2000. **47**(3): p. 344-50.
